# Total Oxidant and Antioxidant Capacity of Gingival Crevicular Fluid and Saliva in Patients with Periodontitis: Review and Clinical Study

**DOI:** 10.3390/antiox9050450

**Published:** 2020-05-23

**Authors:** Joanna Toczewska, Mateusz Maciejczyk, Tomasz Konopka, Anna Zalewska

**Affiliations:** 1 Department of Periodontology, Wrocław Medical University, 50-425 Wroclaw, Poland; tomasz.konopka@umed.wroc.pl; 2 Department of Hygiene, Epidemiology and Ergonomics, Medical University of Bialystok, 15-222 Bialystok, Poland; mat.maciejczyk@gmail.com; 3 Experimental Dentistry Laboratory, Medical University of Bialystok, 15-222 Bialystok, Poland; azalewska426@gmail.com

**Keywords:** periodontal disease, saliva, gingival crevicular fluid, salivary diagnostics

## Abstract

Periodontitis is inextricably linked to oxidative-reductive (redox) imbalance. However, little is still known about the resultant ability to scavenge oxygen free radicals in saliva and gingival crevicular fluid in patients with periodontitis. The multitude of enzymatic and non-enzymatic antioxidants and their synergistic effects cause an interest in the evaluation of the total antioxidative capacity. Thus, our study aimed to evaluate the total oxidative and antioxidative activity of gingival crevicular fluid and saliva in the periodontitis, as well as to relate these biomarkers to clinical indices of periodontopathy. Additionally, by calculating the oxidative stress index (OSI), the intensity of redox disturbances was also evaluated. Fifty-eight periodontitis patients were included in the study and divided into two subgroups depending on the severity of the disease. In the non-stimulated/stimulated saliva as well as a gingival crevicular fluid of the study group, we found significantly higher OSI and total oxidant status (TOS) as well as lower total antioxidant capacity (TAC). However, the ability to reduce iron ions (FRAP) was significantly lower only in stimulated and non-stimulated saliva of patients with periodontitis. The examined parameters correlated with the periodontium’s clinical condition, which indicates the exacerbation of the inflammatory process. However, TAC, TOS, OSI, and FRAP did not differentiate individual stages of periodontitis.

## 1. Introduction

Periodontitis is an oral multifactorial disorder leading to progressive destruction of the periodontal attachment apparatus. Interventional and cohort studies indicate that periodontitis may be an independent risk factor for diabetes, atherosclerotic cardiovascular diseases (ACVDs) and low birth weight in infants; whereas cross-sectional and case-control studies show periodontitis as a possible risk factor for metabolic syndrome, chronic renal failure, rheumatoid arthritis and neurodegenerative diseases [1]. A new classification of periodontal diseases, developed in 2017 during the World Workshop on the Classification of Periodontal and Peri-implant Diseases and Conditions, underlines the association between periodontal diseases and systemic diseases affecting the host immune response. This classification aims to identify individual patients in a targeted manner and to indicate those who require greater efforts to control periodontal disease. However, it hinders the extrapolation of previous research results to the periodontal diagnoses described nowadays [2,3].

Oxidative stress plays a vital role in the etiopathogenesis of all systemic diseases. Periodontitis, like any chronic inflammatory disease, is also inextricably linked with oxidative-reductive imbalance [4]. Indeed, stimulation of neutrophils by periopathogens leads to a “respiratory burst” resulting in increased formation of reactive oxygen (ROS) and nitrogen (RNS) species mainly hypochlorous acid, superoxide anion, and hydrogen peroxide. Many studies have shown that oxidative stress is directly responsible for the degradation of extracellular matrix components of periodontal tissue, including collagen, elastin, proteoglycans, and glycosaminoglycans (e.g., hyaluronic acid) [4]. This leads to the destruction of the periodontal attachment apparatus [5,6,7]. Nevertheless, oxidative stress also initiates/promotes the inflammatory response in periodontitis. Under the influence of ROS, an increase in cytokine production and growth factors (e.g., interleukin-6 (IL-6) and -8 (IL-8), tumor necrosis factor α (TNF-α), and nuclear factor κB (NFκB)) has been shown [7]. Indeed, in patients with periodontal disease, the activity of NADPH oxidase (NOX) increases, which not only enhances the production of free radicals but is also an important source of pro-inflammatory cytokines [8]. The NADPH oxidase NOX2 plays a role in periodontal pathologies. Oxidative stress also leads to the release of lysosomal enzymes responsible for local tissue destruction. Interestingly, the results of recent studies indicate that in periodontitis patients with comorbidities, salivary redox disturbances are exacerbated. In cases with periodontitis and coronary heart disease (CKD) it has been demonstrated enhanced levels of salivary and serum malondialdehyde (lipid peroxidation marker) as well as asymmetric dimethylarginine (ADMA, an endogenous inhibitor of nitric oxide) compared to healthy subjects and CHD cases. This was accompanied by a decrease in vitamin C, one of the most important oral antioxidants, whereas C-reactive protein (CRP) has been found to be a significant predictor of enhanced malondialdehyde and ADMA levels [9,10,11].

It is believed that the disruption of the antioxidant barrier is directly responsible for oxidative/nitrosative modifications of cell components [12]. Indeed, when the antioxidant reserves are exhausted, there is no scavenging of oxygen free radicals nor their neutralization. However, in the course of periodontal disease, both an increase/decrease in activity and concentrations of several free radical scavengers were observed [4,7]. The multitude of enzymatic and non-enzymatic antioxidants as well as their synergistic effects cause an interest in the evaluation of the total oxidative and antioxidative capacity. Indeed, sometimes much more information is provided by the total free radical scavenging capacity than the evaluation of a concentration for each antioxidant separately. Although this is some simplification, it allows a less complicated comparison of the intensity level of redox imbalance. Spectrophotometric, electrochemical, and chromatographic methods are used for this purpose [13,14,15]. Although these methods have a similar measuring principle, the contribution of individual antioxidants to the total antioxidant potential varies. It is therefore necessary to evaluate several parameters characterizing the total antioxidant potential of the body [16]. The recent research results indicate that the antioxidant/oxidant capacity of saliva is used in the diagnosis of such systemic diseases as chronic renal disease, hypertension, chronic heart disease, or psoriasis [17,18,19,20,21]. Nevertheless, reports on the use of these biomarkers in the diagnosis of periodontopathy are incomplete and contradictory. There is also no research comparing (non-)stimulated saliva and gingival crevicular fluid (GCF).

Therefore, our research aimed to evaluate the total oxidative and antioxidative activity of gingival crevicular fluid and saliva in periodontitis, as well as to relate these biomarkers to clinical markers of periodontopathy (according to the most recent classification of periodontal diseases). Additionally, by calculating the oxidative stress index (OSI), the intensity level of redox imbalance was evaluated.

## 2. Materials and Methods

### 2.1. Patients

The research was carried out between February 2018 and March 2019. 251 patients with periodontitis and healthy controls were selected among those who attended the Academic Dental Polyclinic (Department of Periodontology), Wroclaw Medical University, Poland. All patients were Caucasian Poles aged between 20 and 55 years. After screening, 156 patients were excluded from the experiment because they declined to participate (*n* = 22) or did not meet the inclusion criteria (*n* = 113). Therefore, 58 patients with periodontitis and 58 healthy controls were finally enrolled.

The diagnosis was established based on a clinical examination according to the currently accepted definition of periodontitis [2,3]. The study group was divided into two subgroups depending on the periodontitis severity [3]: stage III (34 persons) and stage IV (24 persons). The control group included 29 subjects with healthy periodontium (BOP < 10%, PD ≤ 3 mm), matched with age and sex to the study group.

The exclusion criteria for both the study and control group included: age below 20 and above 55 years, oxidative stress-related systemic diseases, pregnancy, periodontal treatment—less than a year before the study, taking any medications and dietary supplements for 6 months before the study, a number of teeth below 15, a lesion on the oral mucosa, current smoking.

The study was approved by the Bioethics Committee of the Medical University of Wroclaw (approval number: KB-559/2018). Written informed consent was obtained from each patient.

### 2.2. Sample Collection

The research material was mixed saliva (both non-stimulated and stimulated) as well as gingival crevicular fluid (GCF).

Saliva collection was done in a separate room, always between 8 and 10 a.m., using the spitting method. Saliva was collected after a 5-min adaptation period, in a sitting position, with head slightly tilted down and minimizing lip and face movements. After rinsing the mouth three times with distillate water, saliva was spat into a sterile Falcon^®^ (BD Biosciences, San Jose, CA, USA) tube placed in an ice container [22]. Non-stimulated saliva was collected up to a maximum volume of 5 mL in no more time than 10 min. Stimulated saliva was collected for 5 min. Salivation was stimulated by dropping 10 μL of 2% citric acid per tongue every 30 s [23]. Saliva volume was measured using an automatic pipette with an accuracy of 0.1 mL. The salivary flow rate was calculated by dividing the saliva volume by the time necessary for its secretion (mL/min). Immediately after collection, the samples were centrifuged (5000× *g*, 20 min, 4 °C). The supernatant fluid was retained for testing and an antioxidant (10 μL of 0.5 M-butylated hydroxytoluene (BHT)/1 mL of saliva) was added to all samples. Then, the saliva and GCF were frozen at −80 °C [20].

GCF was collected from the clinically deepest periodontal pockets. The area was isolated from saliva access using cotton dental rollers and then dried with compressed air. Gingival fluid was collected using PerioPaper Strips^®^ (Oraflow, New York, NY, USA). The strips contaminated with blood or saliva were discarded. Before and after collecting, strips were placed in Eppendorf^®^ tubes (Eppendorf, Warszawa, Polska) and weighed on an analytical balance to determine the volume of gum fracture fluid. Antioxidant (10 μL 0.5 M BHT/ 1 mL of GCF) was added [20] and samples were frozen at −80 °C.

### 2.3. Clinical Examination

The clinical condition of the periodontium was evaluated based on a clinical periodontal examination. The examination was conducted in artificial lighting; a periodontal probe (calibrated every 1 mm) and a mouth mirror were used. The following variables were evaluated: on two tooth surfaces—modified PI index by O’Leary et al. [24]; in interdental spaces—API index by Lange et al. [25]; Bleeding on Probing (BoP) by Ainamo and Bay [26], examined at 6 points of each tooth (presence of bleeding was recorded up to 30 s after probing); Papillary Bleeding Index (PBI) by Saxer and Mühlemann [27]; pocket depth (PD), examined at 6 points of each tooth; clinical attachment level (CAL)—at 6 points of each tooth. Based on PD and CAL measurements, the following values were calculated: average PD for all teeth, measured at 6 points of each tooth; average interproximal PD for all teeth, measured at 4 points of each tooth; a number of pockets with PD > 5 mm; on interproximal surfaces—a percentage of teeth with CAL ≥ 5 mm; a percentage of regions with CAL > 0 mm; average CAL on interproximal surfaces from CAL > 3 mm regions. Tooth mobility was assessed using an electronic device—Periotest^®^ (Medif, Warszawa, Polska) (maximum and mean value from the measurements made). The clinical examination was conducted by one calibrated researcher. In 20 patients, the intra- and inter-rater reliability between the examiner and another experienced periodontologist were assessed. The reliability of all indices was >0.91.

### 2.4. Redox Assays

On the day of the assays, saliva and GCF were slowly thawed at 4 °C. For GCF extraction, PerioPaper Strips^®^ (Oraflow, New York, NY, USA) were placed in Eppendorf^®^ (Eppendorf, Warszawa, Polska) tubes containing 0.02 M phosphate-buffered saline (PBS) (1 strip/500 μL PBS pH 7.0). The samples were mixed for 30 s in a vortex mixer and then centrifuged (4 °C, 3000× *g*, 5 min) [12,28].

The gingival crevicular fluid was used for all determinations on the same day. The samples containing saliva and GCF were mixed with a vortex mixer immediately prior to determining.

Total oxidative status (TOS) was determined using the colorimetric method described by Erel [14]. In this method, Fe^2+^ ions are oxidized to Fe^3+^ ions in the presence of oxidants contained in the sample. Next, Fe^3+^ ions detection is carried out with xylenol orange. The TOS concentration was calculated from the calibration curve of hydrogen peroxide. TOS determination was carried out in triplicates.

TAC was measured using the colorimetric method described by Erel [29]. The method’s principle is based on the measurement of the ability to neutralize 2,2-azino-bis-(3-ethylbenzothiazoline-6-sulfonate cationic radical (ABTS^•+^) under the influence of antioxidants contained in the sample. Variations in the absorbance of the ABTS^•+^ solution are measured at 660 nm. To determine TAC concentration, the samples (5 μL) were incubated with 200 μL of 0.4 M acetate buffer at pH 5.8. Subsequently, 20 μL of ABTS^•+^ solution was added in 30 mm acetate buffer at pH 3.6. The samples were incubated and absorbance was measured at the wavelength of 660 nm. The TAC concentration was calculated based on the standard curve for Trolox (6-hydroxy-2,5,7,8-tetramethyl-chroman-2-carboxylic acid) and presented in µm Trolox/mg of total protein. TAC determination was carried out in triplicates.

The oxidative stress index (OSI) is presented as the quotient of TOS to TAC and expressed in % [14,30].

The ability to reduce iron ions (FRAP) was determined by the colorimetric method based on the reduction of Fe^3+^-TPTZ complex (iron-2,4,6-tripyridyl-s-triazine complex) to Fe^2+^-TPTZ under the influence of antioxidants contained in the test sample. The maximum absorption of the resulting complex occurs at the wavelength of 593 nm. To determine FRAP concentration, 75 μL of the test sample was incubated with 2.25 mL of a tenfold diluted solution containing 10 mm TPTZ, 20 mm iron chloride (3+) in 300 mm acetate buffer at pH 3.6. After incubating the samples, the absorbance was measured at the wavelength of 593 nm. FRAP concentration was calculated based on the standard curve for iron sulfate and presented in µm/mg of total protein. FRAP determination was carried out in triplicates.

Total protein levels were determined colorimetrically using a commercially available PIERCE BCA protein analysis kit from Thermo Scientific (Rockford, IL, USA). The absorbance of the samples was measured at 562 nm and total protein levels were read from the standard curve for bovine serum albumin (BSA). Total protein levels are expressed in μg/mL.

### 2.5. Statistical Analysis

The results were standardized to mg of total protein. The statistical analysis found no normal distribution of all variables, which was verified by the Shapiro-Wilk test. Mann-Whitney U test were used to compare two groups, while ANOVA Kruskal–Wallis with Tukey’s post-hoc test for three groups. Spearman’s test was used in the analysis of covariation. The adopted statistical significance threshold was *p* ≤ 0.05, while in the correlation analysis—*p* ≤ 0.02. Multiplicity adjusted *p*-value was also calculated. The analysis was carried out using the statistical software package Statistica 13.1. (StatSoft, Wrocław, Poland).

The number of patients was determined based on our pilot study involving 15 patients and 15 healthy controls. The test power was assumed at 0.9 (2-sided significance level of 0.05) and the TAC assay was used for the calculation (sample size online calculator ClinCalc). The minimum number of patients was 26.

## 3. Results

General and periodontal data of patients are presented in Table 1.

### 3.1. Total Oxidant Status (TOS)

**Figure 1:**
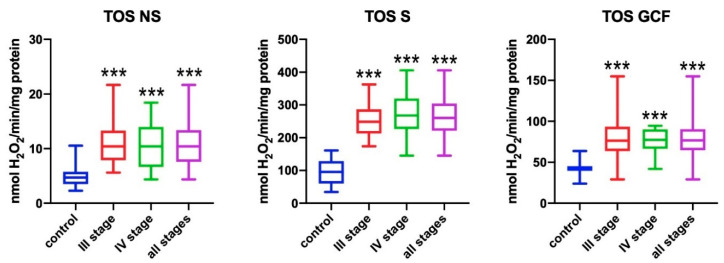
Concentrations of total oxidative status (TOS) in non-stimulated saliva (NS), stimulated saliva (S), and gingival crevicular fluid (GCF) in all patient groups. *** *p* < 0.001 vs. control group.

TOS was significantly higher in the non-stimulated saliva of all patients with periodontitis when compared to healthy subjects (*p* < 0.001) (Figure 1). In patients with stage III and IV of periodontitis, the total oxidant status was significantly higher when compared to healthy subjects (*p* < 0.001). TOS in the stimulated saliva of all subjects with periodontitis was significantly higher when compared to healthy persons (*p* < 0.001). Stage III and IV patients showed significantly higher TOS in stimulated saliva than subjects from the control group (*p* < 0.001). In all subjects with periodontitis, TOS in gingival crevicular fluid (GCF) was significantly higher when compared to healthy subjects (*p* < 0.001). In stage III and IV patients, significantly higher TOS in GCF was observed when compared to subjects from the control group (*p* < 0.001).

### 3.2. Total Antioxidant Capacity (TAC)

**Figure 2:**
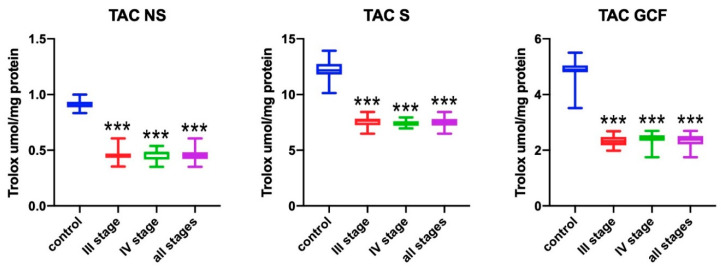
Concentrations of total antioxidant capacity (TAC) in non-stimulated saliva (NS), stimulated saliva (S), and gingival crevicular fluid (GCF) in all patient groups. *** *p* < 0.001 vs. control group.

The total antioxidant capacity in the non-stimulated saliva of all patients was significantly higher when compared to healthy subjects (*p* < 0.001) (Figure 2). Patients with stage III and IV of periodontitis had a significantly lower TAC when compared to healthy subjects (*p* < 0.001). Similarly, TAC in the stimulated saliva of all patients with periodontitis was significantly lower when compared to healthy subjects (*p* < 0.001). Stage III and IV patients had significantly lower TAC than subjects from the control group (*p* < 0.001). In all subjects with periodontitis, TAC in GCF was significantly higher when compared to healthy subjects (*p* < 0.001). Patients with stage III and IV of periodontitis showed significantly lower TAC in GCF when compared to the control group (*p* < 0.001).

### 3.3. Oxidative Stress Index (OSI)

**Figure 3:**
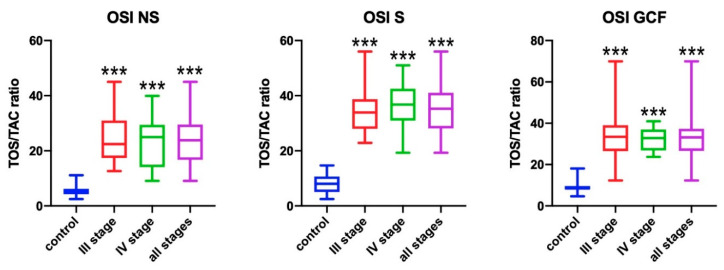
Concentrations of oxidative stress index (OSI) in non-stimulated saliva (NS), stimulated saliva (S), and gingival crevicular fluid (GCF) in all patient groups. *** *p* < 0.001 vs. control group.

The OSI was significantly higher in the non-stimulated saliva of all patients with periodontitis comparing to healthy people (*p* < 0.001) (Figure 3). Patients with stage III and IV of the disease had significantly higher OSI in the non-stimulated saliva compared to the control group (*p* < 0.001). The same relation was found for all study groups (stage III, IV, and all both for stimulated saliva (*p* < 0.001) and gingival crevicular fluid (*p* < 0.001).

### 3.4. FRAP

**Figure 4:**
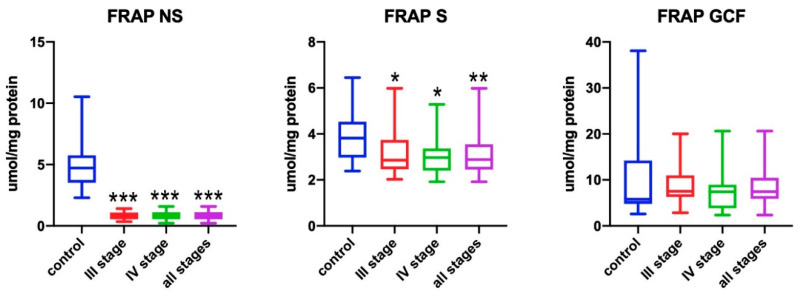
Concentrations of ferric ion reducing antioxidant power (FRAP) in non-stimulated saliva (NS), stimulated saliva (S), and gingival crevicular fluid (GCF) in all patient groups. * *p* < 0.05, ** *p* < 0.01, *** *p* < 0.001 vs. control group.

The ability to reduce iron ions in the non-stimulated saliva of all patients with periodontitis was significantly lower when compared to healthy subjects (*p* < 0.001) (Figure 4). Patients with stage III and IV of periodontitis had significantly lower FRAP when compared to healthy subjects (*p* < 0.001). FRAP in the stimulated saliva of all patients with periodontitis was significantly lower when compared to healthy subjects (*p* = 0.001). It was found that stage III patients had significantly lower FRAP in stimulated saliva than subjects from the control group (*p* = 0.026), whereas stage IV patients had even lower mean FRAP values when compared to periodontally healthy subjects (*p* = 0.013). There were no statistically significant differences in the reduction capacity of iron ions in GCF between the patient groups analyzed.

### 3.5. Correlations

The total oxidant status in non-stimulated saliva significantly correlated positively with a number of teeth—in the whole group (*R* = 0.404; *p* = 0.003) and in stage IV (*R* = 0.62, *p* = 0.002). TOS in stimulated saliva significantly correlated with the number of preserved teeth in the group of patients with stage IV periodontitis only (*R* = 0.517; *p* = 0.019). The TOS values in GCF showed only a weakly significant covariation in the whole group of patients with periodontitis, with mean CAL on the interproximal surfaces from regions with 3-mm loss at least (*R* = 0.323; *p* = 0.019). The total antioxidant capacity (TAC) in non-stimulated saliva did not correlate significantly with any clinical parameter; in stimulated saliva, only a weakly negative covariation was found between the whole group of subjects with periodontitis and PI index (*R* = −0.394; *p* = 0.004). Regarding TAC in GCF, there was a weakly significant correlation with age in the whole group of patients (*R* = 0.329; *p* = 0.015) and a slightly more strongly significant correlation with the number of pockets with PD above 5 mm (*R* = 0.359; *p* = 0.008). The OSI in non-stimulated saliva significantly correlated with the number of teeth only—in the whole group (*R* = 0.37; *p* = 0.008) and in stage IV (*R* = 0.563, *p* = 0.006). OSI in stimulated saliva and GCF did not show any significant covariation with clinical parameters. In both types of saliva and GCF, FRAP did not correlate significantly with clinical variables.

## 4. Discussion

Despite the increasing knowledge of the etiopathogenesis of inflammatory periodontal diseases, the diagnostics and classification of these diseases are almost exclusively based on traditional clinical evaluation. Since periodontal diagnosis is largely subjective and retrospective, it is not surprising that biochemical biomarkers of periodontopathy are constantly being sought. The indicators evaluated in GCF/saliva could be useful in objectivizing the diagnosis and determining the severity of the disease, as well as in evaluating treatment results.

We have shown a decrease in the total antioxidant potential (↓TAC, ↓FRAP) and an increase in total oxidant activity (↑TOS, ↑OSI). This study is the first one where TOS/TAC were compared to GCF and both stimulated and non-stimulated saliva in patients with periodontitis. We have not found any correlations between the biomarkers measured in NWS, SWS, and GCF. Although the examined parameters correlated with the periodontium’s clinical condition, TAC, TOS, OSI, and FRAP did not differentiate individual stages of periodontitis.

TOS expresses the total amount of oxidants in a test sample. In our study, we found significantly higher concentrations of TOS in GCF and both types of saliva in the periodontitis when compared to the clinically healthy periodontium. In stimulated saliva, mean TOS values were the highest for the other two fluids tested, as well as the highest TOS values in the most advanced stage of the disease. This fact is not surprising since the parotid gland secreting stimulated saliva is the most important source of free radicals among all salivary glands [31,32]. These observations are consistent with the findings of Wei et al. [33] and Baltacioğlu et al. [34]. In the former research, patients with CP (chronic periodontitis) showed significantly higher mean TOS values in order of CGF, blood serum, and non-stimulated saliva when compared to the control group without any pathological changes in the periodontium. In the latter one, the higher levels of TOS were also significantly higher in the order of serum and non-stimulated saliva of patients with periodontitis (significantly higher as regards aggressive periodontitis than chronic one) when compared to the control group comprising people with clinically healthy periodontium. Zhang et al. [35] did not find any significant difference in salivary TOS concentration between patients with periodontitis and the control group; however, the high percentage of active nicotinists in both groups could significantly affect the result of this observation. All these studies confirm significantly higher ROS concentrations in GCF, saliva, and blood serum during periodontitis once again. Our patients with periodontitis showed a positively significant correlation between TOS in non-stimulated saliva and the number of teeth only (an understandable covariation of the number of “giving” sites of hydrogen peroxide and its concentration) and, in stage IV, between TOS activity in GCF and the number of regions with at least 3-mm clinical loss of attachment on interproximal surfaces. This is in some opposition to the findings of Baltacioğlu et al. [34], who showed strong correlations between salivary/serum TOS levels and clinical parameters of periodontal condition (PI, GI, PD, and CAL), as well as to the findings of Wei et al. [33], who found weaker but also significant correlations between TOS in GCF/saliva/serum and the same clinical markers of periodontopathy. Additionally, no link between salivary TOS and the amount of periopathogens in saliva (*P. gingivalis*, *T. denticola*, *T. forsythia*, *A. actinomycemecomitans*, and *F. nucleatum*) has been found [35]. The analysis of own research results does not confirm the suggestion of other authors [33,34] that the TOS levels in non-stimulated saliva would be a good marker, stratifying the severity of periodontitis.

Table 2 compares the most significant studies on the evaluation of the total antioxidant capacity of GCF, saliva, and serum (plasma) in the periodontitis [34,35,36,37,38,39,40,41,42,43,44,45,46,47,48,49,50,51,52,53,54,55].

In our research, we found a significant reduction in the exclusively endogenous TAC in GCF of periodontal pockets when compared to gingival fissures, and the extent of this reduction did not coincide with the division into stages and grades of periodontitis. The point of reference in the literature is the study by Becerika et al. [52], who, using the same methodology of TAC evaluation, also found its reduction, but it did not reach the level of statistical significance. This was probably due to the significantly smaller group investigated in Turkish observation when compared to our own (20 vs. 60 subjects). When comparing the TAC evaluation, identically methodological studies should be compared to each other, as each method prefers other non-enzymatic antioxidants in the overall capacity assessment. In the TEAC method by Erel, the activity of ROS scavengers—in the form of proteins containing thiol groups at the expense of uric acid—is measured to a greater extent [56]. Similarly, the TAC activity spectrum (in GCF, 75% of it depends on thiol proteins) means the enhanced chemiluminescence (ECL) method used by Chapple et al. [37,40,41,42]; they also found a significant reduction in the TAC levels in GCF from periodontal pockets when compared to the control group. On the other hand, in our study, after applying the FRAP method, there was no significant difference in the antioxidant capacity of GCF between the study and control groups. This method is only slightly sensitive to the activity of thiol proteins and prefers uric acid as the mainly assessed antioxidant [56]; therefore, in such a compartment, its concentration is not the most important element in ROS inactivation. Becerik et al. [52] stated, however, that a significant decrease in the antioxidant capacity (FRAP) of gingival crevicular fluid in patients with periodontitis as well as quite incomprehensible inverse covariations with the clinical parameters of periodontal condition —in particular with the pocket depth and the clinical attachment level. These differences probably resulted from the different methodologies of determining the final FRAP concentration (in our study, they were referred to total protein in GCF, whereas in the Turkish study the GCF volume was determined using Periotron 8000). That is why all these above-mentioned studies indicate a local significant reduction in the antioxidant capacity in the periodontitis. The positively significant correlation between the TAC in gingival crevicular fluid and the number of periodontal pockets above 5 mm, demonstrated throughout our study group, proves either a negative effect of this inhibition on the clinical condition of the periodontium or vice versa. The clarification of the nature of this dysfunction is provided by interventional studies, in which the impact of standard non-surgical periodontal treatment on TAC in GCF of periodontal pockets was evaluated. The first study of this type was conducted by Chapple et al. [41] and showed that such treatment significantly improved TAC in gingival crevicular fluid, measured with ECL, to the control group level. In another two studies, Turkish authors [57,58] proved that such treatment significantly improved TAC in the gingival crevicular fluid by means of the TEAC method by Erel. This discovery concerned deep periodontal pockets, and that effect lasted for at least 6 weeks. At the same time, it was indicated that certain general conditions, such as smoking or familial Mediterranean fever (FMF), made such an improvement impossible. In other words, both TAC evaluation methods proved that suppression of the antioxidant activity in pocket compartments results from the inflammatory and immunological process, rather than predisposes to it. In our study, by means of a comprehensive evaluation of the pro- and antioxidant effect, determination of the oxidative stress index has become possible. This indicator is one of the direct parameters of prevalence and measurement of oxidative stress intensity in many systemic diseases [59]. Due to the fact that TOS was significantly increased and TAC in compartment fluid of the periodontal pockets was lowered when compared to GCF of periodontally healthy subjects, the local OSI value in periodontitis was significantly higher (approx. 4 times).

The total antioxidant capacity of the salivary compartment is significantly different from that of the gingival crevicular fluid. Firstly, the impact of exogenous, non-enzymatic antioxidants contained in food is significant and difficult to control. Secondly, salivary glands, especially during stimulated salivary secretion (in our study: a significantly higher TAC when compared to GCF and non-stimulated saliva), may be a source of many non-enzymatic antioxidants, including, in particular, uric acid, ascorbate, and drugs with such properties, e.g., allopurinol. It was found that both in saliva and urine, uric acid (70%) and ascorbate have the highest percentage share in TAC [60]. In clinical-control studies, it was found that the consistency between salivary TAC and the concentration of uric acid was 75% [60]. In the study carried out by Zhang et al. [35], a multifactorial analysis showed that out of 9 variables only the diagnosis of periodontitis was significantly related to salivary TAC (regardless of age, sex, smoking or semi-quantitative occurrence of 5 periopathogens in saliva). In the vast majority of studies [34,35,39,43,48,50,54], as well as in our study, the salivary TAC profile in patients with periodontitis was significantly reduced in ABTS oxidation method. Only one study [47] did not prove the statistical significance of this difference, although the mean difference between the control and study groups was large (0.59 vs. 0.4 μM). Meta-analysis by Chen et al. [61], involving 4 studies described in Table 2 [34,47,48,53] and 3 others [62,63,64] as well as referring to 556 subjects, showed a significant reduction in the salivary TAC for periodontitis in relation to the control group (*p* = 0.003, inhomogeneity index: 98.3%). In our study of periodontitis, the total antioxidant capacity of saliva (mainly non-stimulated one) was significantly reduced, as also described earlier by Dhotre et al. [62] and Baňasova et al. [50], but only in women. The significant effect of periodontitis on the antioxidant capacity of saliva in the FRAP method was not proved in two determinations [38,53]. Such discrepancy may result from gender. The authors of this method, Benzie and Chung [15], have already described the significantly higher antioxidant capacity of plasma in women, which they explained with higher concentrations of uric acid (UA). In the context of testing the antioxidant capacity in periodontal patients, it seems that a better way to obtain saliva is non-stimulated saliva—less exposed to the fluctuations of its various components during stimulation of large salivary glands, as several authors have pointed out [38,53]. After using other methods to evaluate the total antioxidant capacity of saliva in periodontopathies, the results significantly differed from those presented above. After using the ECL method, no effect of periodontal diagnosis on TAC in stimulated and non-stimulated saliva was observed [40]. After using the TRAP method in chronic and aggressive periodontitis, the significantly higher antioxidant capacity of non-stimulated saliva was described in relation to periodontally healthy control groups, suitable for these diagnoses [51]. This result explains the sensitivity of this method to the concentration of antioxidants—proteins and polyphenols were responsible for 57%, whereas ascorbate, UA, and tyrosine were responsible for 37% in plasma [65], which can be with high probability extrapolated to saliva. If analytical methods sensitive to the detection of uric acid are used, a significant reduction in the total antioxidant capacity is observed—mainly in the non-stimulated saliva of patients with periodontitis. It is probably a transfer of this antioxidant deficit from the pockets themselves, together with the combination of several confounding factors (e.g., exogenous supply of polyphenols), weakening this effect. In our patients, such a dependence was confirmed in stimulated saliva with plaque index. A significant increase in OSI in periodontitis was also observed in our study and the salivary compartment as well (the highest mean value was found in stimulated saliva, in the most advanced stage of the disease), which confirms earlier observations [34,54]. Thus, quite intense oxidative stress associated with periodontitis is transferred from the pockets to the saliva, including the weakening of non-stimulated saliva. This oxidative stress is primarily caused by a significant increase in the total oxidant status (a very high positive correlation in the whole study group between OSI and TOS in non-stimulated saliva, with no OSI-TAC relationship and no positive OSI-teeth number relationship, i.e., the number of pockets emitting ROS).

It is suggested that shifting the salivary/GCF redox balance in favor of the oxidative reactions (↓TAC, ↓FRAP, ↑TOS, ↑OSI) predisposes to oxidative damage to proteins, lipids, and DNA in the periodontal tissue. It was shown that cell oxidation products stimulate the synthesis of arachidonic acid derivatives (especially prostaglandin (Pg)E2), pro-inflammatory cytokines (e.g., IL-1, IL-6) as well as cell adhesion molecules (e.g., intercellular adhesion molecule 1, ICAM-1). Macrophages and fibroblasts accumulate in such altered periodontium. These cells are also the source of numerous cytokines, metalloproteinases (MMPs), and proteolytic enzymes, which leads to progressive destruction of the periodontal attachment apparatus [4,6].

The studies of the total antioxidant capacity of plasma or serum in patients with periodontitis are a complement of great importance to these determinations carried out at the site of the disease (tissue, gingival crevicular fluid) and saliva. This is because they indicate the potential for the interaction of periodontal oxidative stress with the systemic inflammatory process and its endpoints. In this case, as in saliva, the tests sensitive to the detection of antioxidant properties of uric acid and ascorbates would be the most effective. The use of oxidation methods (by Miller, Erel) or ABTS cationic radical reduction methods quite clearly demonstrated a significant decrease in TAC in peripheral blood, in the periodontitis [34,42,44,46,49,54,55,63]. Only two observations have not found any effect of periodontal diagnosis on the change of TAC in blood serum or plasma [52,58]. Out of 9 studies of TAC in plasma, serum, and blood, a meta-analysis by Liu et al. [66], involving 248 subjects in the study group and 238 in the control group, proved a significant decrease in the total antioxidant capacity in periodontitis (*p* = 0.000, inhomogeneity index: 95.5%). This result should be approached with caution since 5 studies have been incorrectly included due to different methodologies of the TAC test and the occurrence of exclusionary general conditions. Despite a significant decrease in the total antioxidant activity in peripheral blood of patients with periodontitis, it does not seem possible that the magnitude of the related oxidative stress might have systemic implications. Alas, we did not assess in our study the total antioxidant capacity of plasma/blood serum in patients with periodontitis, which makes it impossible to conclude central redox homeostasis, thus it constitutes a limitation of the study.

Initially, the diagnosis of periodontal diseases was based exclusively on the examination of GCF taken with the use of filters from periodontal pockets. This technique, however, is very time-consuming as well as technically demanding, and the filters are easily contaminated with blood or bacterial plaque. The ease and noninvasiveness of the collection make saliva a biological fluid that can be applied in the diagnosis of oral diseases. Nonetheless, as our studies have shown, none of the evaluated biomarkers in saliva/GCF differentiates the stages of periodontitis. TAC, TOS, and OSI also correlate weakly with clinical periodontal markers. Although salivary antioxidant/oxidant status is used in the diagnosis of many systemic diseases (chronic renal disease, hypertension, psoriasis), it should be remembered that changes in salivary redox homeostasis may result from changes at the systemic level [9,17,18,19,67]. Indeed, salivary redox biomarkers faithfully reflect their plasma/blood serum content as well as they correlate with classical disease progression indicators (e.g., creatinine in chronic renal disease or diastolic pressure in hypertension). Low diagnostic usefulness of redox biomarkers in periodontal diseases may result from the fact that the oral cavity is constantly exposed to many environmental factors, such as air pollution, tobacco smoke, food, and microorganisms [68]. Dental materials and dental procedures performed within the oral cavity are also of great importance. They may destabilize local redox homeostasis, making saliva/GCF useless in the diagnosis of oral diseases. Furthermore, there is a lack of reference values for the redox salivary/GCF biomarkers assessed, which makes it difficult to compare the results obtained in different centers.

## 5. Conclusions

In conclusion, the significant reduction of the total antioxidant capacity was found both in GCF and saliva of patients with periodontitis, most likely due to the chronic inflammatory process. Such a condition may predispose to oxidative damage to proteins, lipids, and DNA and may cause progressive destruction of the periodontal attachment apparatus. However, TAC, TOS, OSI, and FRAP did not differentiate individual stages of periodontitis and, therefore, cannot be used for routine periodontal diagnosis. It is necessary to carry out further research on a greater number of patients with periodontitis.

## Figures and Tables

**Table 1:** General and periodontal data. API—approximal plaque index; BoP—bleeding on probing; CAL—clinical attachment level; PD—probing depth; PI—plaque index.

Parameter		Control			Stage III			Stage IV			All Stages		
		Median	Min	Max	Median	Min	Max	Median	Min	Max	Median	Min	Max
Age		39	20	55	44	20	55	45	29	55	45	20	55
sex	Women	17 (59%)			16 (47%)			13 (54%)			29 (50%)		
	Men	12 (41%)			18 (53%)			11 (46%)			29 (50%)		
Unstimulated saliva flow (mL/min)		0.4	0.2	1	0.4	0.1	1	0.45	0.1	1.3	0.4	0.1	1.3
Stimulated saliva flow (mL/min)		1.6	0.4	3.4	1.4	0.3	3	1.4	0.6	3	1.4	0.3	3
Protein in unstimulated saliva (μg/mL)		589.51	300.45	1101	827.95 *	481.23	1387	839.62 *	23.48	1847.1	833.79 *	23.48	1847.1
Protein in stimulated saliva (μg/mL)		598.98	235.74	946.29	610.32	28.91	926.41	634.78	43.74	811.92	634.78	28.91	926.41
Protein in gingival fluid (μg/mL)		30.94	8.44	91.66	130.43 *	36.8	336.97	134.24 *	45.5	445.64	131.13 *	36.8	445.64
Number of teeth		27	19	28	28	24	28	23*	15	28	26	15	28
PI		20	0	79	43 *	9	100	43.5 *	0	100	43.5 *	0	100
API		32	7	68	65 *	29	100	86.5 *	22	100	72.5 *	22	100
BoP		10	0.7	26	41 *	4	100	61 *	17	100	46.5 *	4	100
PD		1.7	1.2	2.3	3.15 *	2.1	5.3	4.1 *	2.7	5.4	3.5 *	2.1	5.4
Mean CAL > 0		2.1	1	5.2	4.95 *	2.4	8.1	6.05*	3	10.1	5.4*	2.4	10.1

* *p* < 0.05 vs. control group.

**Table 2:** Comparison of studies on total antioxidant capacity in periodontitis.

Author, Year, and Country	Fluid Method	Study Group Size and Age	*p* for Perio	Other Data
Chapple et al. 1997 [36], Great Britain	NS Serum **TAC**—Chemiluminescence	CP—18 (>35) HP—16 (>35)	Saliva ↓ *p* < 0.01 Serum n.s.	
Chapple et al. 2002 [37], Great Britain	GCF Plasma **TAC**—Chemiluminescence	P—10 (mean 46,1) HP—10 (mean 46,9)	GCF ↓ *p* < 0.015 Plasma n.s.	
Sculley and Langley-Evans 2003 [38], Great Britain	NS **FRAP**—TPTZ Benzie, 1996	AgP—46 (mean 59,6) HP—46 (mean 60,3)	FRAP n.s.	
Diab-Ladki et al. 2003 [39], Libia	S **TAC**—ABTS Miller, 1997	AgP—17 (30–45) HP—20 (30–45)	↓ *p* < 0.05 (40%)	
Brock et al. [40] 2004, Great Britain	S NS GCF Serum **TAC**—Chemiluminescence	CP—17 (mean 43,5) HP—27 (mean 44,7)	GCF ↓ *p* < 0.001 NS, S, serum n.s.	No significant correlation in GCF TAC-PD, TAC in saliva lower than GCF *p* < 0.0005.
Chapple et al. [41] 2007, Great Britain	GCF Plasma **TAC**—Chemiluminescence	CP—35 (32–61)		Significant raise in TAC in GCF after periodontal treatment *p* < 0.001, plasma n.s.
Konopka et al. [42] 2007, Poland	Serum **TAC**—ABTS Re, 1999	CP—30 (mean 44,9) AgP—26 (mean 31,5) HP—25 (mean 33,2)	CP and AgP ↓ *p* = 0.000, ↓ in CP/AgP	No significant correlation with clinical status.
Su et al. [43] 2009, Canada	NS **TAC**—ABTS Miller, 1997	P—58 (mean 52,3) HP—234 (mean 45,4)	↓ *p* < 0.0001	Significant correlation with perio disease and CPITN
Abou Sulaiman et al. [44] 2010, Siria	Plasma **TAC**—ABTS Erel, 2004	CP—30 (mean 41) HP—30 (mean 34)	↓ *p* < 0.001	No significant correlation with clinical status; significant raise after periodontal treatment.
Dhotre et al. [45] 2012, India	Serum **FRAP**—TPTZ Benzie, 1996	P—25 (no data) HP—25 (no data)	↓ *p* < 0.001	
Konuganti et al. [46], 2012, India	Blood **TAC**—NBT	CP—15 (18–40) HP—15 (18–40)	↓ *p* < 0.001	
Novakovic et al. [47] 2014, Serbia	NS **TAC**—ABTS	CP—21 (mean 39,1) HP—21 (mean 35,2)	TAC-n.s.	No significant correlation with clinical status.
Miricescu et al. [48], 2014, Romania	NS **TAC**—ABTS	CP—20 (mean 51,3) HP—20 (mean 18,6)	↓ *p* < 0.05	
Baltacioğlu et al. [34] 2014, Turkey	NS Serum **TAC**—ABTS Erel, 2004 **OSI**—Erel, 2004	CP—33 (>40) AgP—35 (18–40) HP—30 (no data)	Saliva, serum ↓ TAC *p* = 0.001 ↑ OSI *p* = 0.001	Significant correlation: TAC in saliva and serum with PI, GI, PD and CAL. Significant positive correlation with OSI.
Thomas et al. [49] 2014, India	Serum **TAC**—No data	CP—25 (no data) HP—25 (no data)	↓ *p* < 0,001	
Baňasova et al. [50] 2015, Slovakia	NS **TAC**—ABTS Erel, 2004 **FRAP**—TPTZ, Benzie, 1996	CP—23 (mean 43) HP—19 (mean 39,1)	↓TAC in women *p* < 0.01 ↓ FRAP in women	Significant positive correlation TAC and FRAP with a low value of PD and CAL.
Acquier et al. [51] 2016, Argentina	NS **TRAP**—Lissi and Vargas	CP—20 (mean 37,4) AgP—20 (mean 19,5) HP—20 (17–40)	CP and AgP ↑ *p* < 0.001 ↑ CP/AgP	No significant correlation with clinical status.
Becerik et al. [52] 2017, Turkey	GCF Plasma **TAC**—ABTS Erel, 2004 **FRAP**—TPTZ, Benzie, 1996	CP—20 (mean 43,1) HP—20 (mean 38,4)	GCF TAC n.s. plasma ↓ FRAP TAC n.s.	Significant correlation in GCF: FRAP-PI, PBI, PD, and CAL; positive significant correlation with plasma TAC-PD FRAP-CAL
Ahmadi-Motamayel et al. [53] 2017, Iran	NS Serum **FRAP**—Riviere	CP—55 (30–50) HP—55 (30–50)	Saliva and serum n.s.	
Tripathi et al. [54] 2018, India	NS Serum **TAC**—ELISA **OSI**—ABTS Erel, 2004	CP—40 (>18) AgP—40 (18–40) HP—40 (no data)	Saliva ↓ TAC *p* = 0.04 ↑ OSI (in CP) *p* = 0.01 Serum ↓ TAC *p* = 0.03 ↑ OSI *p* = 0.02	↑ OSI saliva in CP *p* = 0.01 ↑ OSI serum in CP and AgP *p* = 0.02
Narenda et al. [55] 2018, India	GCF Serum **TAC**—ABTS Miller, 1997	CP—46 (mean 47,1) AgP—32 (mean 25,7) HP—50 (mean 36,6)	GCF in AgP ↓ *p* < 0.001 Serum in AgP ↓ *p* < 0.001	
Own study	GCF NS S **TAC, OSI**—ABTS Erel, 2004 **FRAP**—TPTZ, Benzie, 1996	P—60 (mean 43,6) HP—30 (mean 40,3)	GCF ↓TAC *p* < 0.001 ↑OSI *p* < 0.001 FRAP n.s. Saliva ↓TAC *p* < 0.001 ↑OSI *p* < 0.001 ↓ FRAP *p* < 0.001	For significant p positive correlations between TAC in GCF—PD > 5 mm, negative correlation with TAC in stimulated saliva with PI; for stage IV negative correlation between OSI in NS—PI.

NS—non-stimulated saliva; S—stimulated saliva; GCF—gingival crevicular fluid; TAC—total antioxidant capacity; FRAP—ferric ion reducing antioxidant power; OSI—oxidative stress index; P—periodontitis; CP—chronic periodontitis; AgP—aggressive periodontitis; HP—healthy patients; n.s.—statistically non-significant; CPITN—Community Periodontal Index of Treatment Needs; PI—plaque index; PBI—papilla bleeding index; GI—gingival index; PD—pocket depth; CAL—clinical attachment level.
